# Nocturnal ambush predators and their potential impact on flower‐visiting moths

**DOI:** 10.1002/ecy.3482

**Published:** 2021-10-16

**Authors:** Kota Sakagami, Daichi Funamoto, Shinji Sugiura

**Affiliations:** ^1^ Graduate School of Agricultural Science Kobe University 1–1 Rokkodaicho Nada, Kobe Hyogo 657–8501 Japan

**Keywords:** mantis, mantisfly, Mantodea, Neuroptera, pollinators, predation, Scutigeromorpha, settling moths

Ambush predators such as spiders affect the flower‐visiting behavior of diurnal pollinators such as bees (Dukas 2001, Dukas and Morse 2003), potentially causing diurnal pollinators to avoid flowers where ambush predators wait (Dukas 2001). Thus, ambush predators can diminish the reproductive success of flowering plants (Gonçalves‐Souza et al. 2008). Although nocturnal insects such as moths are important pollinators of many flowering plants (Hahn and Brühl 2016), the impact of ambush predators on nocturnal pollinators remains unclear.

On 19 September 2018, we found a praying mantis, *Tenodera sinensis* (Mantodea: Mantidae), eating a moth, *Sarcopolia illoba* (Lepidoptera: Noctuidae), on flowers of *Eupatorium lindleyanum* (Asteraceae) at night in Hyogo, Japan (Fig. 1A). This suggests that ambush predators such as praying mantises prey on nocturnal pollinators, as they do on diurnal pollinators. Nocturnal moths on flowers are reportedly eaten by spiders (Morse 1983), mantises (Delf and Harris 1964), and bats (Martins and Johnson 2013). However, few studies have quantified the abundance of nocturnal predators on flowers and their predation pressures on nocturnal pollinators in the field.

**Fig. 1:**
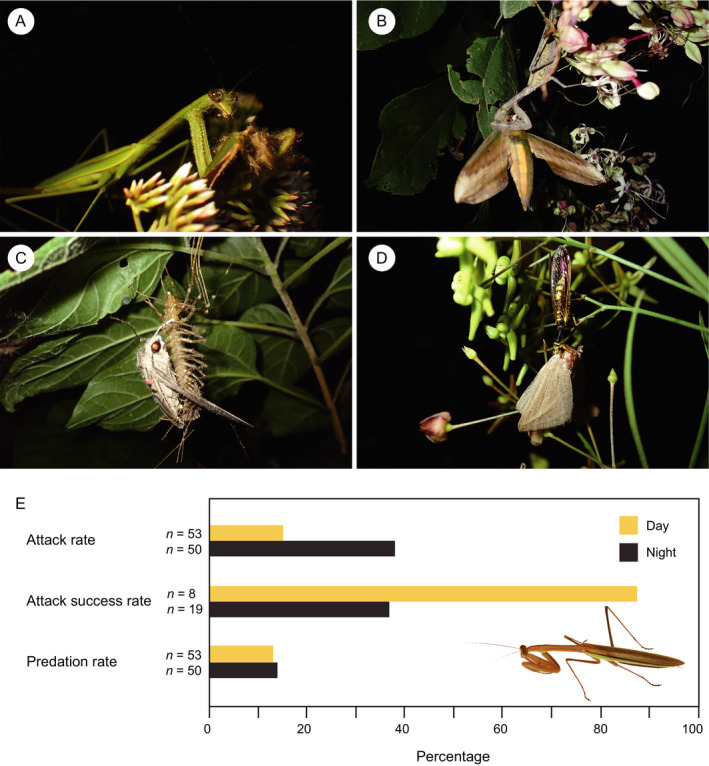
Nocturnal ambush predators preying on flower‐visiting moths. (A) A mantis (*Tenodera sinensis*) eating a settling moth (*Sarcopolia illoba*) on *Eupatorium lindleyanum*. (B) A mantis (*Hierodula patellifera*) eating a hawkmoth (*Theretra japonica*) on *Clerodendrum trichotomum*. (C) A house centipede (*Thereuopoda clunifera*) eating a hawkmoth (*Agrius convolvuli*) on *C*. *trichotomum*. (D) A mantisfly (*Austroclimaciella quadrituberculata*) eating a settling moth (*Rhynchina cramboides*) on *Vincetoxicum pycnostelma*. (E) The rates of attack, attack success, and predation on flower visitors by praying mantises during the day and at night (Appendix S1: Table S7). Attack rate = (numbers of visitors attacked by mantises)/(numbers of visitors within mantis attack range). Attack success rate = (numbers of visitors eaten by mantises)/(numbers of visitors attacked by mantises). Predation rate = (numbers of visitors eaten)/(numbers of visitors within mantis attack range).

To estimate the abundance of nocturnal predators on flowers, we counted the numbers of ambush predators on nine plant species at night during July–October 2019 in western Honshu, Japan (Table 1, Appendix S1: Methods S1, Table S1). These plants attract nocturnal flower visitors, such as moths (Appendix S1: Methods S1). Field observations were conducted at eight sites (grassland, wetland, garden, and forest) where flowering individuals of focal plant species were abundant (Appendix S1: Table S1). The predators were quantified using “snapshot” counts (cf., Garbuzov and Ratnieks 2014), in which the number of predators on flowers or other plant organs <150 mm from the flowers was determined nearly instantaneously by eye (total observation time 16.1 h). Whether each predator ate flower visitors was also recorded. The numbers of ambush predators were counted on six of the nine plant species during the day (total observation time 8.1 h) to compare the abundances of nocturnal and diurnal predators (Appendix S1: Methods S2, S3, Table S1).

**Table 1:** Frequencies of ambush predators and flower visitors on nine plant species.

Flowering plant species	Predators per 100 units†				Flower visitors per unit per h†				Unit‡
	Daytime		Night		Daytime		Night		
*Vincetoxicum pycnostelma*	–		23.3	(1.8)	–		0.50	(1.0)	inflorescence§
*Eupatorium lindleyanum*	0.35	(1.3)	0.66	(1.8)	1.59	(1.0)	0.49	(1.0)	inflorescence§
*Adenophora triphylla*	0	(3.2)	0.13	(5.4)	0	(1.0)	0.43	(1.0)	inflorescence§
*Abelia* × *grandiflora*	0	(0.3)	0.05	(0.3)	0.93	(0.5)	0.19	(1.0)	flower
*Trichosanthes cucumeroides*	–		3.23	(0.2)	–		2.00	(0.5)	flower
*Lespedeza cyrtobotrya*	0.01	(1.3)	0.06	(1.3)	1.29	(1.0)	0.04	(1.0)	flower
*Clerodendrum trichotomum*	0.50	(1.0)	3.60	(2.0)	3.07	(1.0)	0.91	(1.0)	inflorescence§
*Lythrum anceps*	0.17	(1.0)	0.47	(2.0)	1.47	(1.0)	0.51	(1.0)	inflorescence§
*Hemerocallis citrina*	–		1.20	(1.3)	–		3.50	(1.0)	flower

† Numbers of predators per 100 units and flower visitors per unit per h are shown. The total observation hours are in parentheses. A dash indicates that diurnal observations were not conducted because these plant species bloom mainly at night.

‡ Units for counting predators and flower visitors.

§ Because the flowers were too numerous to count individually, we counted the numbers of predators and flower visitors per inflorescence.

Diverse ambush predators were found on the flowers of nine plant species including praying mantises (three species; Mantodea), mantisflies (one species; Neuroptera), fishing spiders (one species; Araneae), a house centipede (one species; Scutigeromorpha), and a centipede (one species; Scolopendromorpha) (Appendix S1: Table S2). Mantises (*Hierodula patellifera*, *T*. *sinensis*, and *Tenodera angustipennis*) comprised 92.3% of the ambush predators on flowers (Appendix S1: Table S2). On the flowers of six plant species that bloom throughout the day, these ambush predators were no less abundant at night than during the day (Table 1). Furthermore, we found that mantises, mantisflies, a house centipede, and a fishing spider preyed on flower‐visiting moths on seven plant species at night (Fig. 1B–D, Appendix S1: Table S3, Video S1). To our knowledge, this is the first study to document predation on nocturnal flower visitors by mantisflies and house centipedes.

To estimate how often predators potentially encounter flower visitors, we also examined the frequency and species composition of visitors to flowers without predators of nine plant species from July to September of 2019 (total observation times: day, 5.5 h; night, 8.5 h; Appendix S1: Methods S4, Tables S4, S5). The rate of flower visitation by insects at night did not differ greatly from that during the day in six plant species (Table 1). However, the flower visitors differed greatly between day and night; diurnal flower visitors were mainly bees (54.7%), hoverflies (9.0%), and butterflies (34.5%), whereas nocturnal flower visitors were exclusively moths (99.7%; Appendix S1: Table S6).

To compare the predation pressures of ambush predators on nocturnal flower visitors with those on diurnal visitors, we observed the foraging behavior of mantises (*T*. *sinensis* and *T*. *angustipennis*) on *E*. *lindleyanum* flowers using night‐shot video cameras with infrared light in September and October of 2020 (total observation times: day, 45.9 h; night, 44.4 h; Appendix S1: Methods S4). Praying mantises recognized the insects that approached or landed on *E*. *lindleyanum* flowers. Mantises used their raptorial forelegs to attack 15.1% and 38.0%, respectively, of diurnal and nocturnal visitors within attack range (i.e., <50 mm in front of the mantises; Fig. 1E, Appendix S1: Table S7). Of the attacking mantises, 87.5% and 36.8% successfully captured diurnal and nocturnal visitors, respectively (Fig. 1E, Appendix S1: Table S7, Video S1). Consequently, 13.2% and 14.0% of diurnal and nocturnal visitors were eaten by mantises (Fig. 1E, Appendix S1: Tables S7, S8). Mantises compensate for a lower capture success at night by attacking visitors more frequently, resulting in nearly equal predation pressures on nocturnal flower visitors as on diurnal visitors. This equal susceptibility of nocturnal and diurnal pollinators raises the question of whether nocturnal pollinators (i.e., moths) have also evolved to avoid predators on flowers.

Nocturnal moths are categorized into two functional groups based on their morphology and behavior: hawkmoths (Sphingidae) hover while feeding on nectar, whereas settling moths (e.g., Noctuoidea, Pyraloidea, and Geometroidea) land on flowers while feeding on nectar (e.g., Atwater 2013). In this study, a long‐tongued hawkmoth *Agrius convolvuli* (Sphingidae) was observed to perform conspicuous lateral swinging movements like a pendulum while hovering and feeding on nectar of *Hemerocallis citrina* (Video S2). Wasserthal (1998) postulated that the swing‐hovering behavior of long‐tongued hawkmoths while feeding on nectar plays an important role in decreasing predation risk by ambush predators, such as huntsman spiders. Although the swing‐hovering behavior of settling moths has not been reported, settling moths may also be able to avoid ambush predators on flowers. For example, plusiine moths such as *Ctenoplusia albostriata* (Noctuidae) were frequently observed to flutter their wings while landing on flowers to feed on floral nectar (Video S3; Sakagami and Sugiura 2019); this behavior might enable the moths to escape predators quickly. In addition, the hairs/scales and behavior of the moths might also help them avoid mantis attacks, because 70.6% of attacked lepidopterans escaped from the mantises’ raptorial forelegs (Appendix S1: Table S9). Furthermore, settling moths were frequently observed to fly around flowers before landing on them (Video S3), suggesting that settling moths use visual or olfactory cues to recognize and avoid flowers with ambush predators. Field experiments are needed to test this hypothesis. Specifically, the flower‐visiting behavior of settling moths should be observed on flowers with and without predators. If settling moths can recognize ambush predators on flowers, they should avoid the flowers with predators more frequently than those without predators. Consequently, the mere presence of nocturnal predators may reduce the fruit or seed set.

## Supporting information

Appendix S1

Video S1

Video S2

Video S3

Video S1Legend

Video S2Legend

Video S3Legend

## Data Availability

Data (Sakagami et al. 2021) available in Figshare: https://doi.org/10.6084/m9.figshare.13596197.
