# Supplementary material for: Nocturnal ambush predators and their potential impact on flower‐visiting moths
Source: Ecology. 2021 Oct 16;102(11):e03482. doi: 10.1002/ecy.3482 (PMC9286552; doi:10.1002/ecy.3482)
Supplement: Supplementary file 1 — Appendix S1 [file ECY-102-0-s001.pdf]

Supporting Information. Sakagami, K., D. Funamoto, and S. Sugiura. 2021. Nocturnal ambush predators and their potential impact on flower-visiting moths. *Ecology*. https://doi.org/10.1002/ecy.3482

APPENDIX S1

METHODS

S1. Abundance of predators on flowers

To estimate the abundance of nocturnal predators on flowers, we counted the numbers of ambush predators on the flowers and other organs <150 mm from the flowers or inflorescences of nine plant species at night (19:30–24:30) during July–October 2019 in western Honshu, Japan (Table S1). Flowers of these plant species reportedly attract nocturnal flower visitors, such as moths (Ikenoue 1999, Tanaka 2009, Nakahama et al. 2013). We quantified the predators using "snapshot" counts, in which the number of predators was determined nearly instantaneously by eye (cf., Garbuzov and Ratnieks 2014). Predators concealed within/under flowers were not included in this study. A researcher counted the numbers of predators on 3–871 flowers or inflorescences and other plant organs <150 mm from the flowers or inflorescences of five plant species (*Vincetoxicum pycnostelma*, *Adenophora triphylla*, *Trichosanthes cucumeroides*, *Clerodendrum trichotomum*, and *Hemerocallis citrina*). However, the flowers or inflorescences were too numerous to count individually in the other four plant species (*Eupatorium lindleyanum*, *Abelia* × *grandiflora*, *Lespedeza cyrtobotrya*, and *Lythrum anceps*). Therefore, a researcher counted the numbers of flowers or inflorescences in a quadrat (0.5 × 0.5 m^2). For each plant species, 16–131 quadrats were set on flower patches, and the numbers of predators in each quadrat were counted. Similarly, 2–10 quadrats were set, and the numbers of flowers or inflorescences in each quadrat were counted. We multiplied the mean numbers of flowers or inflorescences per quadrat by the total numbers of quadrats (16–131) to estimate the total numbers of flowers or inflorescences. Thus, the numbers of predators per flower or inflorescence were estimated in the four plant species.

In six plant species (*E. lindleyanum*, *Ad. triphylla*, *Ab.* × *grandiflora*, *Le. cyrtobotrya*, *C. trichotomum*, and *Ly. anceps*) that bloom during the day, we counted or estimated the numbers of predators per flower or inflorescence during daytime (10:30–18:00) to compare the densities of ambush predators between day and night (Table S1).

S2. Abundance of flower visitors

To estimate how often ambush predators potentially encounter flower visitors, we determined the rate of flower visitation by insects to the nine plant species at the same sites as the numbers of visits by insects per flower or inflorescence per hour (Table S4). Flowers without ambush predators were observed from July to September of 2019. Flower visitors to five plant species (*E. lindleyanum*, *Ab.* × *grandiflora*, *Le. cyrtobotrya*, *C. trichotomum*, and *Ly. anceps*) were observed directly by a researcher for 0.5–1.0 h during daytime (12:00–16:00) and for 1.0 h at night (18:30–20:00). Flower visitors to *Ad. triphylla* were observed directly by a researcher for 1.0 h during daytime (13:40–14:40) and recorded using seven night-shot video cameras with infrared light (DVSA10FHDIR, Kenko) for 1.0 h at night (18:40–19:40). Flower visitors to *V. pycnostelma* were recorded using two video cameras for 1 h at night (18:40–19:40). Flowers of two plant species (*H. citrina* and *T. cucumeroides*) were observed directly by a researcher for 0.5–1.0 h at night (18:40–19:40).

S3. Floral traits

To examine the floral traits of each plant species, we measured the floral tube length, nectar volume, and nectar sugar concentration from July to October of 2019 (Table S5). Floral tube length (the distance between bottom and opening of the flower) was measured to the nearest 0.01 mm using digital calipers (CD-15AX, Mitsutoyo; 4–27 flowers per plant species). Nectar volume was measured using 1-, 2-, 5-, and 10-μL microcapillary tubes (minicaps, Hirschmann; 1–26 flowers per plant species). The nectar sugar concentration was measured using a hand-held refractometer (0–50% Brix scale, 45-81 Eclipse, Bellingham and Stanley; 1–23 flowers per plant species).

S4. Predation pressures on flower visitors by mantises

To estimate the predation pressures of ambush predators on flower visitors, we recorded the foraging behaviors of praying mantises (*T. angustipennis* and *T. sinensis*) on *E. lindleyanum* flowers using night-shot video cameras with infrared light (DVSA10FHDIR, Kenko) during the day (12:00–17:15) and at night (17:30–21:30) from September to October of 2020 at Miki-shi, Hyogo (Table S1). There were no artificial lights at the study site. The sunset time ranged from 17:42 to 18:24 during the study period. The sunset times were obtained from the National Astronomical Observatory of Japan (https://www.nao.ac.jp/), the moon phases from the Time and Date AS (https://www.timeanddate.com/), and weather details from the Japan Meteorological Agency (https://www.jma.go.jp/jma/indexe.html). The sunset times, moon phases, and weather details are included in the raw data file. When mantises were found on *E.*

lindleyanum flowers and other organs <100 mm from the flowers, their behavior was recorded using night-shot video cameras. The behaviors of 46 mantises were recorded (total observation times: day, 45.9 h; night, 44.4 h). Of these mantises, nine did not encounter flower visitors during the video recording. Mantises that shot their forelegs toward flower visitors were considered to have attacked them. The attack range of mantises was determined based on the attacking behavior of the recorded mantises (e.g., <50 mm in front of the mantises). Data on 37 mantises that encountered flower visitors within attack range (53 daytime and 50 nighttime encounters) were analyzed (Fig. 1E, Table S7). Mantises that caught and ate flower visitors were considered to have preyed on them.

To compare mantis predation on nocturnal and diurnal visitors, we constructed a generalized linear mixed model with a binomial error distribution and logit link (Table S8). Whether a mantis captured a flower visitor within attack range (1/0) was used as a binary response variable. Diurnal or nocturnal visitors were used as explanatory variables. Individuals of mantises were included as a random effect. This analysis was performed with the lme4 package 1.1.0 (Bates et al. 2015) of R ver. 3.3.2 (R Core Team 2016).

Literature Cited

Bates, D., M. Mächler, B. M. Bolker, and S. C. Walker. 2015. Fitting linear mixed-effects models using lme4. Journal of Statistical Software 67:1–48.

Garbuzov, M., and F. L. W. Ratnieks. 2014. Quantifying variation among garden plants in attractiveness to bees and other flower-visiting insects. Functional Ecology 28:364–374.

Ikenoue, T. 1999. Records of moths attracted to flowers in the eastern part of Yamaguchi Prefecture. Yugato Supplement 7:1–123. (in Japanese).

Nakahama, N., R. Miura, and T. Tominaga. 2013. Preliminary observations of insect visitation to flowers of *Vincetoxicum pycnostelma* (Apocynaceae: Asclepiadoideae), an endangered species in Japan. Journal of Entomological Science 48:151–160.

R Core Team. 2016. R: a language and environment for statistical computing. Vienna, Austria: R Foundation for Statistical Computing.

Tanaka, H. 2009. The handbook of flowers pollinated by insects. Bunichi Sogo Shuppan, Tokyo. (in Japanese).

TABLE S1. Field observations of ambush predators on flowers of nine plant species.

Plant family	Plant species	Site<br>Latitude, longitude, altitude	Habitat	Period	Method†	Numbers of days	Total numbers of flowers or inflorescences	Date
Apocynaceae	*Vincetoxicum pycnostelma*	Mine-shi, Yamaguchi	Grassland	Daytime	–	–	–	–
		34°15′N, 131°19′E, 294 m		Night	Direct	7	30	4–10 Sep. 2019
Asteraceae	*Eupatorium lindleyanum*	Miki-shi, Hyogo	Wetland	Daytime	Quadrat	2	4867	20, 24 Sep. 2019
		34°53′N, 135°04′E, 138 m		Night	Quadrat	3	5429	15, 21, 23 Sep. 2019
		Nishi-ku, Kobe-shi, Hyogo	Wetland	Daytime	Quadrat	1	333	27 Sep. 2019
		34°45′N, 135°00′E, 138 m		Night	Quadrat	1	333	26 Sep. 2019
Campanulaceae	*Adenophora triphylla*	Mine-shi, Yamaguchi	Grassland	Daytime	Direct	7	4928	5–11 Sep. 2019
		34°15′N, 131°19′E, 294 m		Night	Direct	8	4629	4–11 Sep. 2019
Caprifoliaceae	*Abelia × grandiflora*	Kasai-shi, Hyogo	Grassland	Daytime	Quadrat	1	4767	30 Aug. 2019
		34°54′N, 134°53′E, 61 m		Night	Quadrat	2	9534	29, 30 Aug. 2019
Cucurbitaceae	*Trichosanthes cucumeroides*	Nishi-ku, Kobe-shi, Hyogo	Garden	Daytime	–	–	–	–
		34°45′N, 135°02′E, 94 m		Night	Direct	2	155	6, 12 Aug. 2019
Fabaceae	*Lespedeza cyrtobotrya*	Mine-shi, Yamaguchi	Grassland	Daytime	Quadrat	4	7196	8–11 Sep. 2019
		34°15′N, 131°19′E, 294 m		Night	Quadrat	4	7196	7–10 Sep. 2019
Lamiaceae	*Clerodendrum trichotomum*	Kita-ku, Kobe-shi, Hyogo	Forest	Daytime	Direct	2	1205	25 Aug., 2 Sep. 2019
		34°43′N, 135°05′E, 256 m		Night	Direct	4	1556	21, 26, 31 Aug., 1 Sep. 2019
Lythraceae	*Lythrum anceps*	Ono-shi, Hyogo	Wetland	Daytime	Quadrat	2	3440	25 Aug., 2 Sep. 2019
		34°50′N, 134°53′E, 49 m		Night	Quadrat	3	5160	22, 30, 31 Aug. 2019
Xanthorrhoeaceae	*Hemerocallis citrina*	Nishi-ku, Kobe-shi, Hyogo	Wetland	Daytime	–	–	–	–
		34°45′N, 135°00′E, 132 m		Night	Direct	5	581	30 Jul., 1,2, 4, 5 Aug. 2019

†Direct: the numbers of predators were counted on flowers or inflorescences; quadrat: the numbers of predators were counted in quadrats (0.5 × 0.5 m^2) in flower patches; –: diurnal observations were not made because these species mainly bloomed at night.

TABLE S2. Ambush predator species and abundance on flowers of nine plant species.

Plant		Predator			
Family	Species	Order	Species	Number of individuals†	
				Daytime	Night
Apocynaceae	*Vincetoxicum pycnostelma*	Neuroptera	*Austroclimaciella quadrituberculata*	–	7 (1)
Asteraceae	*Eupatorium lindleyanum*	Mantodea	*Tenodera angustipennis*	8 (0)	13 (1)
			T. sinensis	10 (0)	25 (0)
Campanulaceae	*Adenophora triphylla*	Mantodea	*T. angustipennis*	0 (0)	5 (0)
		Neuroptera	*A. quadrituberculata*	0 (0)	1 (0)
Caprifoliaceae	*Abelia* × *grandiflora*	Mantodea	*Hierodula patellifera*	0 (0)	1 (0)
			T. sinensis	0 (0)	4 (0)
Cucurbitaceae	*Trichosanthes cucumeroides*	Araneae	*Dolomedes sulfureus*	–	3 (0)
		Mantodea	*H. patellifera*	–	2 (0)
Fabaceae	*Lespedeza cyrtobotrya*	Mantodea	*T. angustipennis*	1 (0)	4 (0)
Lamiaceae	*Clerodendrum trichotomum*	Mantodea	*H. patellifera*	6 (0)	53 (1)
			T. sinensis	0 (0)	1 (1)
		Scolopendromorpha	*Scolopendra mutilans*	0 (0)	1 (0)
		Scutigeromorpha	*Thereuopoda clunifera*	0 (0)	1 (1)
Lythraceae	*Lythrum anceps*	Araneae	*D. sulfureus*	1 (0)	0 (0)
		Mantodea	*T. angustipennis*	2 (0)	12 (0)
			T. sinensis	3 (1)	12 (1)
Xanthorrhoeaceae	*Hemerocallis citrina*	Mantodea	*Tenodera* spp.‡	–	7 (0)

†–: Diurnal observations were not conducted because these plant species bloomed mainly at night. The units for predator counts are shown in Table 1. The number of predation events is shown in parentheses.

‡These predators were larvae. Other predators were adults.

TABLE S3. Observations of ambush predators eating flower visitors under field conditions.

Plant		Predator		Period	Prey (flower-visiting insects)			Method†
Family	Species	Order	Species		Order	Family	Species (*n*)	
Apocynaceae	*Vincetoxicum pycnostelma*	Neuroptera	*Austroclimaciella quadrituberculata*	Night	Lepidoptera	Erebidae	*Rhynchina cramboides* (1)	Direct
						Pyralidae	*Endotricha* sp. (2)	Video
Asteraceae	*Eupatorium lindleyanum*	Mantodea	*Tenodera angustipennis*	Daytime	Diptera	Syrphidae	*Eristalis tena* (1)	Video
					Hymenoptera	Scoliidae	*Campsomeris prismatica* (3)	Video
				Night	Diptera	Culicidae	Unidentified (1)	Video
					Lepidoptera	Geometridae	Larentiinae gen. (1)	Video
							Sterrhinae gen. (1)	Video
						Noctuidae	*Pyrrhidivalva sordida* (1)	Direct
						Unidentified	Noctuoidea gen. (1)	Video
						Unidentified	Pyraloidea gen. (1)	Video
						Unidentified	Unidentified (1)	Video
			Tenodera sinensis	Daytime	Diptera	Stratiomyidae	*Stratiomys japonica* (1)	Video
						Unidentified	Brachycera gen. (2)	Video
				Night	Diptera	Culicidae	Unidentified (1)	Video
					Lepidoptera	Noctuidae	*Sarcopolia illoba* (1)	Direct
							Unidentified (1)	Direct
Caprifoliaceae	*Abelia × grandiflora*	Mantodea	*T. sinensis*	Night	Lepidoptera	Unidentified	Unidentified (1)	Direct
Cucurbitaceae	*Trichosanthes cucumeroides*	Araneae	*Dolomedes sulfureus*	Night	Lepidoptera	Sphingidae	*Psilogramma increta* (1)	Video
		Mantodea	*T. sinensis*‡	Night	Lepidoptera	Sphingidae	*Agrius convolvuli* (1)	Direct
Lamiaceae	*Clerodendrum trichotomum*	Mantodea	*Hierodula patellifera*	Night	Lepidoptera	Sphingidae	*Theretra nessus* (1)	Direct
			T. sinensis	Night	Lepidoptera	Unidentified	Pyraloidea gen. (1)	Direct
		Scutigeromorpha	*Thereuopoda clunifera*	Night	Lepidoptera	Sphingidae	*A. convolvuli* (1)	Direct
Lythraceae	*Lythrum anceps*	Mantodea	*T. sinensis*	Daytime	Hymenoptera	Apidae	*Apis mellifera* (1)	Direct
				Night	Lepidoptera	Unidentified	Unidentified (1)	Direct
Xanthorrhoeaceae	*Hemerocallis citrina*	Mantodea	*T. sinensis*‡	Night	Lepidoptera	Sphingidae	*Theretra japonica* (1)	Direct

†Direct: predation events were recorded on flowers or inflorescences by eye; video: predation events were video recorded. All observations of ambush predators eating flower visitors on focal plant species during 2018–2020 under field conditions were included.
‡These predators were larvae. Other predators were adults.

TABLE S4. Field observations of flower visitors to nine plant species.

Plant family	Plant species	Site	Period	Method†	Numbers of hours	Date	Numbers of flowers or inflorescences‡
Apocynaceae	*Vincetoxicum pycnostelma*	Mine-shi, Yamaguchi	Daytime	–	–	–	–
			Night	Video	1	6 Sep. 2019	2
Asteraceae	*Eupatorium lindleyanum*	Miki-shi, Hyogo	Daytime	Direct	1	20 Sep. 2019	70
			Night	Direct	1	15 Sep. 2019	67
Campanulaceae	*Adenophora triphylla*	Mine-shi, Yamaguchi	Daytime	Direct	1	7 Sep. 2019	32
			Night	Video	1	10 Sep. 2019	7
Caprifoliaceae	*Abelia* × *grandiflora*	Kasai-shi, Hyogo	Daytime	Direct	0.5	30 Aug. 2019	472
			Night	Direct	1	30 Aug. 2019	472
Cucurbitaceae	*Trichosanthes cucumeroides*	Nishi-ku, Kobe-shi, Hyogo	Daytime	–	–	–	–
			Night	Direct	0.5	5 Aug. 2019	11
Fabaceae	*Lespedeza cyrtobotrya*	Mine-shi, Yamaguchi	Daytime	Direct	1	8 Sep. 2019	218
			Night	Direct	1	9 Sep. 2019	327
Lamiaceae	*Clerodendrum trichotomum*	Kita-ku, Kobe-shi, Hyogo	Daytime	Direct	1	2 Sep. 2019	29
			Night	Direct	1	1 Sep. 2019	23
Lythraceae	*Lythrum anceps*	Ono-shi, Hyogo	Daytime	Direct	1	2 Sep. 2019	95
			Night	Direct	1	31 Aug. 2019	95
Xanthorrhoeaceae	*Hemerocallis citrina*	Nishi-ku, Kobe-shi, Hyogo	Daytime	–	–	–	–
			Night	Direct	1	16 Jul. 2019	24

†Direct: the numbers of visitors were counted on flowers or inflorescences; video: flower visitors were recorded using videos and then counted; –: diurnal observations were not made because these species mainly bloomed at night.

‡The units for flower visitor counts are shown in Table 1.

TABLE S5. Floral traits of the investigated plants.

Plant		Site	Floral tube length (mm)	Nectar volume (μL)	Sugar concentrations (%)
Family	Species		Mean ± SE (*n*)	Mean ± SE (*n*)	Mean ± SE (*n*)
Apocynaceae	*Vincetoxicum pycnostelma*	Mine-shi, Yamaguchi	1.7 ± 0.3 (4)	0.3 ± 0.1 (4)	15.8 ± 1.6 (2)
Asteraceae	*Eupatorium lindleyanum*	Nishi-ku, Kobe-shi, Hyogo	2.3 ± 0.1(6)	0.1 (1)	30.4 (1)
Campanulaceae	*Adenophora triphylla*	Mine-shi, Yamaguchi	11.4 ± 0.2 (27)	0.8 ± 0.2 (26)	17.9 ± 1.2 (23)
Caprifoliaceae	*Abelia* × *grandiflora*	Nada-ku, Kobe-shi, Hyogo	15.5 ± 0.3 (17)	0.8 ± 0.4 (5)	32.0 ± 7.8 (5)
Cucurbitaceae	*Trichosanthes cucumeroides*	Nishi-ku, Kobe-shi, Hyogo	65.6 ± 5.3 (9)	4.4 ± 2.9 (7)	15.8 ± 2.2 (3)
Fabaceae	*Lespedeza cyrtobotrya*	Mine-shi, Yamaguchi	5.1 ± 0.1 (10)	0.7 ± 0.1 (10)	33.1 ± 1.7 (10)
Lamiaceae	*Clerodendrum trichotomum*	Kita-ku, Kobe-shi, Hyogo	22.2 ± 0.7 (16)	3.2 ± 0.7 (16)	18.0 ± 0.7 (14)
Lythraceae	*Lythrum anceps*	Ono-shi, Hyogo	5.7 ± 0.2 (5)	0.2 (1)	–†
Xanthorrhoeaceae	*Hemerocallis citrina*	Nishi-ku, Kobe-shi, Hyogo	29.6 ± 1.4 (12)	17.9 ± 2.1 (12)	19.1 ± 0.4 (12)

†Sugar concentrations could not be measured because the nectar volume was too small.

TABLE S6. Flower visitors on nine plant species.

Plant family	Plant species	Flower visitors		Number of visits†	
		Order	Group	Daytime	Night
Apocynaceae	*Vincetoxicum pycnostelma*	Lepidoptera	Moths	–	1
Asteraceae	*Eupatorium lindleyanum*	Diptera	Hoverflies	76	0
			Others	1	0
		Hymenoptera	Bees	3	0
			Wasps	2	0
		Lepidoptera	Butterflies	29	0
			Moths	0	32
		Neuroptera	Lacewings	0	1
Campanulaceae	*Adenophora triphylla*	Lepidoptera	Moths	0	3
Caprifoliaceae	*Abelia × grandiflora*	Lepidoptera	Butterflies	220	0
			Moths	0	91
Cucurbitaceae	*Trichosanthes cucumeroides*	Lepidoptera	Moths	–	11
Fabaceae	*Lespedeza cyrtobotrya*	Hymenoptera	Bees	280	0
			Wasps	1	0
		Lepidoptera	Moths	0	14
Lamiaceae	*Clerodendrum trichotomum*	Coleoptera	Beetles	1	0
		Diptera	Others	1	0
		Hymenoptera	Bees	67	0
			Wasps	3	0
		Lepidoptera	Butterflies	17	0
			Moths	0	21
Lythraceae	*Lythrum anceps*	Hymenoptera	Bees	110	0
			Wasps	5	0
		Lepidoptera	Butterflies	24	0
			Moths	1	48
Xanthorrhoeaceae	*Hemerocallis citrina*	Lepidoptera	Moths	–	84

†–: diurnal observations were not made because these species mainly bloomed at night. The units for flower visitor counts are shown in Table 1. Flowers without ambush predators were observed.

TABLE S7. Attacks on and predation of flower visitors by praying mantises.

Period	Visitor behavior	Numbers of visitors within mantis attack range	Numbers of visitors attacked by mantises	Numbers of visitors eaten by mantises	Attack rate (%)†	Attack success rate (%)‡	Predation rate (%)§
Daytime	Flying around flowers	11	1	1	9.1	100.0	9.1
	Landing on flowers	42	7	6	16.7	85.7	14.3
	Subtotal	53	8	7	15.1	87.5	13.2
Night	Flying around flowers	16	6	0	37.5	0	0
	Landing on flowers	34	13	7	38.2	53.8	20.6
	Subtotal	50	19	7	38.0	36.8	14.0

†Attack rate = [numbers of visitors attacked by mantises] / [numbers of visitors within mantis attack range].

‡Attack success rate = [numbers of visitors eaten by mantises] / [numbers of visitors attacked by mantises].

§Predation rate = [numbers of visitors eaten by mantises] / [numbers of visitors within mantis attack range].

TABLE S8. Results of a generalized linear mixed model showing the effect of period (day or night) on mantis predation.

Response valuable	Explanatory variable	Coefficient estimate	SE	*z* value	*P* value
Predation	Intercept	−1.8827	0.4057	−4.641	<0.0001
	Period	0.0674	0.5751	0.117	0.907

TABLE S9. Attack and predation by praying mantises on different flower visitor groups.

Visitor group (insect order)	Visitor behavior	Numbers of visitors within mantis attack range	Numbers of visitors attacked by mantises	Numbers of visitors eaten by mantises	Attack rate (%)†	Attack success rate (%)‡	Predation rate (%)§
Diptera	Flying around flowers	11	2	1	18.2	50	9.1
	Landing on flowers	32	5	5	15.6	100	15.6
	Subtotal	43	7	6	16.3	85.7	14.0
Hymenoptera	Flying around flowers	1	0	0	0	–	0
	Landing on flowers	4	3	3	75	100	75
	Subtotal	5	3	3	60	100	60
Lepidoptera	Flying around flowers	15	5	0	33.3	0	0
	Landing on flowers	40	12	5	30.0	41.7	12.5
	Subtotal	55	17	5	30.9	29.4	9.1

†Attack rate = [numbers of visitors attacked by mantises] / [numbers of visitors within mantis attack range].
‡Attack success rate = [numbers of visitors eaten by mantises] / [numbers of visitors attacked by mantises].
§Predation rate = [numbers of visitors eaten by mantises] / [numbers of visitors within mantis attack range].
