# Supplementary material for: Nocturnal ambush predators and their potential impact on flower‐visiting moths
Source: Ecology. 2021 Oct 16;102(11):e03482. doi: 10.1002/ecy.3482 (PMC9286552; doi:10.1002/ecy.3482)
Supplement: Supplementary file 6 — Video S2Legend [file ECY-102-0-s003.pdf]

Supporting Information. Sakagami, K., D. Funamoto, and S. Sugiura. 2021. Nocturnal ambush predators and their potential impact on flower-visiting moths. *Ecology*. https://doi.org/10.1002/ecy.3482

VIDEO. S2. Swing-hovering behavior of a hawkmoth *Agrius convolvuli*. The hawkmoth hovered and swung while feeding on *Hemerocallis citrina* nectar.
