# Supplementary material for: Nocturnal ambush predators and their potential impact on flower‐visiting moths
Source: Ecology. 2021 Oct 16;102(11):e03482. doi: 10.1002/ecy.3482 (PMC9286552; doi:10.1002/ecy.3482)
Supplement: Supplementary file 7 — Video S3Legend [file ECY-102-0-s004.pdf]

Supporting Information. Sakagami, K., D. Funamoto, and S. Sugiura. 2021. Nocturnal ambush predators and their potential impact on flower-visiting moths. *Ecology*. https://doi.org/10.1002/ecy.3482

VIDEO. S3. Flower-visiting behavior of settling moths. The noctuid moth *Ctenoplusia albostriata* fluttered its wings while landing on flowers to feed on *Adenophora triphylla* nectar. The pyraloid moth flew around the *A*. *triphylla* flower before landing.
